# Supplementary material for: Towards UK poSt Arthroplasty Follow-up rEcommendations (UK SAFE): protocol for an evaluation of the requirements for arthroplasty follow-up, and the production of consensus-based recommendations
Source: BMJ Open. 2019 Jun 25;9(6):e031351. doi: 10.1136/bmjopen-2019-031351 (PMC6597629; doi:10.1136/bmjopen-2019-031351)
Supplement: Supplementary data [file bmjopen-2019-031351supp001.pdf]

Appendix 1

Ovid MEDLINE(R) Epub Ahead of Print, In-Process & Other Non-Indexed Citations, Ovid MEDLINE(R) Daily and Ovid MEDLINE(R) 1946 to Present

Search Strategy:

----

1 Arthroplasty, Replacement, Knee/ (18061)
2 Knee Prosthesis/ (10316)
3 (TKA or TKR or UKR).tw. (9131)
4 Arthroplasty, Replacement, Hip/ (21968)
5 Hip Prosthesis/ (21263)
6 (THA or THR).tw. (30477)
7 or/1-6 (79707)
8 Hip/ (11074)
9 Osteoarthritis, Hip/ (7521)
10 Hip Joint/ (24959)
11 Hip?.tw. (121242)
12 Femur Head/ (8758)
13 Acetabulum/ (9886)
14 ("Femur head*" or "femoral head*" or acetabul*).tw. (27939)
15 "Total joint".tw. (4508)
16 Knee/ (12862)
17 Knee Joint/ (47967)
18 Osteoarthritis, Knee/ (15086)
19 Knee?.tw. (122420)
20 or/8-19 [Knee or Hip joints] (259908)
21 Joint Prosthesis/ (9772)
22 "Prostheses and Implants"/ (43103)
23 (Arthroplast* or replace* or implant* or prosthes* or unicompartment*).tw. (724712)
24 (Surf* or resurf*).tw. (990965)
25 or/21-24 [Arthroplasty] (1680546)
26 and/20,25 (82595)
27 7 or 26 [Hip or Knee Arthoplasty] (113853)
28 Longitudinal studies/ (109550)
29 Prospective studies/ (457925)
30 Time/ or time factors/ (1126356)
31 Follow-up studies/ (586823)
32 Epidemiological Monitoring/ (5660)
33 or/28-32 [Follow-up Studies MeSH] (2051292)
34 Retreatment/ (7424)
35 Reoperation/ (76777)
36 Treatment failure/ (31517)
37 exp Postoperative Complications/ (482686)
38 exp Prosthesis failure/ (25670)
39 or/34-38 [Complications MESH] (555690)
40 Risk factors/ (699996)
41 33 or 39 or 40 [Long term complications or risks MESH] (2963609)
42 *Postoperative Care/ (14831)
43 Postoperative care/mt (9905)
44 Postoperative Period/ (42528)
45 Aftercare/ (7484)
46 or/42-45 [Post Operative Care MeSH] (69319)
47 41 and 46 [Post op follow up MeSH] (29208)
48 Critical Pathways/ (5783)
49 47 or 48 [Post op follow up or pathways MeSH] (34925)

50 (("After care" or aftercare or "after surgery" or "after arthroplas*") adj3 follow-up).tw. (2471)
51 ((Postoperati* or post-operati* or "post surger*" or "post arthroplast*") adj3 follow-up).tw. (13670)
52 ((pathway* or care or treatment* or appointment* or consultation*) adj3 follow-up).tw. (29535)
53 ((Postoperati* or post-operati* or "post surger*" or "post arthroplast*") adj3 (surveillance* or monitor*)).tw. (4101)
54 or/50-53 [Follow-up studies Textword] (49052)
55 (("After care" or aftercare or "after surgery" or "after arthroplas*") adj3 (failur* or reoperat* or re-operat* or readmission or readmit* or revision or revisions)).tw. (691)
56 ((Postoperati* or post-operati* or "post surger*" or "post arthroplast*") adj3 (failure* or reoperat* or re-operat* or readmission or readmit* or revision or revisions)).tw. (5650)
57 ((pathway* or care or treatment* or appointment* or consultation* or follow-up or time or risk*) adj8 (revis* adj2 surgery)).tw. (1505)
58 or/55-57 [Post op complications Textword] (7776)
59 (("After care" or aftercare or "after surgery" or "after arthroplas*") adj3 risk*).tw. (637)
60 ((Postoperati* or post-operati* or "post surger*" or "post arthroplast*") adj3 risk).tw. (12221)
61 or/59-60 [Post op risks Textword] (12801)
62 (("After care" or aftercare or "after surgery" or "after arthroplas*") adj3 pathway*).tw. (93)
63 ((Postoperati* or post-operati* or "post surger*" or "post arthroplast*") adj3 pathway*).tw. (181)
64 (Care pathway* or clinical pathway* or critical pathway*).tw. (6772)
65 or/62-64 [Post op pathways Textword] (6956)
66 54 or 58 or 61 or 65 [Post op follow up Textword] (75424)
67 49 or 66 [Post Op Follow Up] (106039)
68 27 and 67 [TJA Post op follow up] (3634)
