# Supplementary material for: Towards UK poSt Arthroplasty Follow-up rEcommendations (UK SAFE): protocol for an evaluation of the requirements for arthroplasty follow-up, and the production of consensus-based recommendations
Source: BMJ Open. 2019 Jun 25;9(6):e031351. doi: 10.1136/bmjopen-2019-031351 (PMC6597629; doi:10.1136/bmjopen-2019-031351)
Supplement: Author's manuscript [file bmjopen-2019-031351.draft_revisions.pdf]

BMJ Open

BMJ Open is committed to open peer review. As part of this commitment we make the peer review history of every article we publish publicly available.

When an article is published we post the peer reviewers' comments and the authors' responses online. We also post the versions of the paper that were used during peer review. These are the versions that the peer review comments apply to.

The versions of the paper that follow are the versions that were submitted during the peer review process. They are not the versions of record or the final published versions. They should not be cited or distributed as the published version of this manuscript.

BMJ Open is an open access journal and the full, final, typeset and author-corrected version of record of the manuscript is available on our site with no access controls, subscription charges or pay-per-view fees (http://bmjopen.bmj.com).

If you have any questions on BMJ Open's open peer review process please email info.bmjopen@bmj.com

BMJ Open

Towards UK poSt Arthroplasty Follow-up rEcommendations (UK SAFE): protocol for an evaluation of the requirements for arthroplasty follow-up, and the production of consensus-based recommendations

Journal:	*BMJ Open*
Manuscript ID	bmjopen-2019-031351
Article Type:	Protocol
Date Submitted by the Author:	29-Apr-2019

Complete List of Authors:	Czoski Murray, Carolyn; University of Leeds Leeds Institute of Health Sciences<br>Kingsbury, Sarah; University of Leeds Leeds Institute of Rheumatic and Musculoskeletal Medicine, Leeds Institute of Rheumatic and Musculoskeletal Medicine; NIHR Leeds Biomedical Research Centre,<br>Arden, Nigel; University of Oxford Nuffield Department of Orthopaedics Rheumatology and Musculoskeletal Sciences<br>Hewison, Jenny; University of Leeds Leeds Institute of Health Sciences<br>Judge, Andrew; University of Bristol, Musculoskeletal Research Unit, Translational Health Sciences, Bristol Medical School, University of Bristol, Learning and Research Building, Level 1, Southmead Hospital, Southmead, BS10 5NB; MRC Lifecourse Epidemiology Unit<br>Matu, Jamie; University of Leeds Leeds Institute of Health Sciences; University of Leeds Leeds Institute of Rheumatic and Musculoskeletal Medicine<br>O'Shea, Jamie; Manor Park Surgery<br>Pinedo-Villanueva, Rafael; University of Oxford Nuffield Department of Orthopaedics Rheumatology and Musculoskeletal Sciences, Nuffield Department of Orthopaedics Rheumatology and Musculoskeletal Sciences<br>Smith, Lindsay; Weston Area Health NHS Trust, Trauma and Orthopaedics; Weston Area Health NHS Trust<br>Smith, Chris; University of Leeds Leeds Institute of Health Sciences<br>Thomas, Christine; NIHR Leeds Biomedical Research Centre; University of Leeds Leeds Institute of Rheumatic and Musculoskeletal Medicine<br>West, Robert; University of Leeds Leeds Institute of Health Sciences<br>Wright, Judy; University of Leeds Leeds Institute of Health Sciences<br>Conaghan, Philip; University of Leeds, Leeds Institute of Rheumatic and Musculoskeletal Medicine and NIHR Biomedical Research Centre<br>Stone, Martin; NIHR Leeds Biomedical Research Centre; Leeds Teaching Hospitals NHS Trust

Keywords:	Hip < ORTHOPAEDIC & TRAUMA SURGERY, Knee < ORTHOPAEDIC & TRAUMA SURGERY, HEALTH SERVICES ADMINISTRATION & MANAGEMENT

Title: Towards UK poSt Arthroplasty Follow-up rEcommendations (UK SAFE): protocol for an evaluation of the requirements for arthroplasty follow-up, and the production of consensus-based recommendations

Authors names:

Carolyn Czoski Murray[1*], Sarah R Kingsbury[2,3*], Nigel K Arden[4,5], Jenny Hewison[1], Andrew Judge[5,6,7], Jamie Matu[1,2], Jamie O'Shea[8], Rafael Pinedo-Villanueva[4], Lindsay K Smith[9], Chris Smith[1], Christine M Thomas[3], Robert West[1], Judy M Wright[1], Philip G Conaghan[2,3^], Martin Stone[3,10^],

*^These authors contributed equally to this work

Department and institution:

1 Leeds Institute of Health Sciences, University of Leeds, Leeds, UK

2 Leeds Institute of Rheumatic and Musculoskeletal Medicine, University of Leeds, Leeds, UK

3 NIHR Leeds Biomedical Research Centre, Leeds, UK

4 Nuffield Department of Orthopaedics, Rheumatology and Orthopaedic Sciences, University of Oxford, Oxford, UK.

5 MRC Lifecourse Epidemiology Unit, University of Southampton, Southampton.

6 Pharmaco- and Device-Epidemiology Group, Centre for Statistics in Medicine, NDORMS, University of Oxford, Oxford.

7 Translational Health Sciences, University of Bristol, Bristol.

8 Manor Park Surgery, Leeds, UK

9 Faculty of Health & Applied Sciences, University of the West of England, Glenside Campus, Bristol, UK.

10 Leeds Teaching Hospitals NHS Trust, Leeds UK

For peer review only

Corresponding author:

Philip G Conaghan

Leeds Institute of Rheumatic and Musculoskeletal Medicine,

Chapel Allerton Hospital,

Chapeltown Rd,

Leeds LS7 4SA, UK

Phone: +44 113 3924884

Fax: +44 113 3924991

Email: p.conaghan@leeds.ac.uk

Keywords: joint replacement, hip arthroplasty, knee arthroplasty, orthopaedic surgery, surveillance

Dates of study: 01/12/2016-30/11/2019

Word count: 4091

For peer review only

Abstract

Introduction:

Hip and knee arthroplasties have revolutionised the management of degenerative joint diseases and, due to an ageing population, are becoming increasingly common. Follow-up of joint prostheses is to identify problems in symptomatic or asymptomatic patients due to infection, osteolysis, bone loss or potential peri-prosthetic fracture, enabling timely intervention to prevent catastrophic failure at a later date. Early revision is usually more straight-forward surgically and less traumatic for the patient. However, routine long-term follow-up is costly and requires considerable clinical time. Therefore, some centres in the UK have curtailed this aspect of primary hip and knee arthroplasty services, doing so without an evidence-base that such disinvestment is clinically- or cost-effective.

Methods:

Given the timeline from joint replacement to revision, conducting a randomised controlled trial (RCT) to determine potential consequences of disinvestment in hip and knee arthroplasty follow-up is not feasible. Furthermore the low revision rates of modern prostheses, less than 10% at 10 years, would necessitate thousands of patients to adequately power such a study. The huge variation in follow-up practice across the UK also limits the generalisability of an RCT. This study will therefore use a mixed-methods approach to examine the requirements for arthroplasty follow-up and produce evidence- and consensus-based recommendations as to how, when and on whom follow-up should be conducted. Four interconnected work packages will be completed: 1) a systematic literature review; 2a) analysis of routinely-collected NHS data from five national datasets to understand when and which patients present for revision surgery; 2b) prospective data regarding how patients currently present for revision surgery; 3) economic modelling to simulate long-term costs and quality-adjusted life years associated with different follow-up care models; 4) a Delphi-consensus process, involving all stakeholders, to develop a policy document which includes a stratification algorithm to determine appropriate follow-up care for an individual patient.

For peer review only

Article Summary

Strengths and limitations of this study

- Our mixed-methods approach allows us to address a question that would not be feasible to answer with a randomised controlled trial.

- Our study will capture data from a mixture of teaching hospitals, district general hospitals and hospitals with a special interest in joint replacement and with a geographical spread, increasing the generalisability of our results.

- Our economic model will be populated with routinely-collected NHS data of patients attending primary and hospital care in the UK, ensuring that our analysis is based upon actual patient use of services, outcomes such as health-related quality of life, and costs to the NHS.

- Whilst our analysis is based on data sources that reflect clinical practice in England only, we believe key cost-effectiveness findings are likely to be informative for decision-making in the whole of the UK.

For peer review only

Introduction

Arguably, total hip arthroplasty (THA) and total knee arthroplasty (TKA) are the most successful surgical interventions performed in modern times. Due to an ageing population, and an obesity epidemic, hip and knee replacement procedures increase annually, rising from less than 20,000/year in the UK in 1978 to around 200,000 /year in 2017.[1] The current follow-up requirements are estimated at 500,000–1,000,000 annual outpatient attendances. With limitless resources every patient undergoing a joint arthroplasty would incur routine lifetime follow-up. The rationale for follow-up is to ensure timely detection of complications or arthroplasty failure, such as aseptic loosening, osteolysis, and potential peri-prosthetic fracture. The cost of revision for aseptic loosening is 35% lower than that for peri-prosthetic fractures, and has a lower incidence of complications which impact recovery.[2] However, whilst routine long-term follow-up of joint prostheses may support timely revision for patients with asymptomatic complications, improving long-term health outcomes, it is also costly both clinically and financially.

Orthopaedic services are already one of the poorest performers across the National Health Service (NHS) by failing to meet waiting list targets, with an estimated 8,000 orthopaedic NHS breaches each month.[3] With a rapidly aging population and medical advances that mean less stringent criteria for surgery eligibility,[4] there is no sign that demand will recede in coming years and orthopaedic services will soon reach breaking point. To reduce the burden on orthopaedic services, evidence-based consensus guidelines are required to establish how, when and on whom follow-up should be conducted.

British Hip Society (BHS) and British Orthopaedic Association (BOA) guidelines recommend outpatient follow-up at 1 and 7 years, and every 3 years thereafter for Orthopaedic Data Evaluation Panel 10A (ODEP-10A) implants, with more frequent follow-up for novel implants.[5] However, recent work revealed considerable diversity across the UK in arthroplasty follow-up pathways, in timing, how follow-up is conducted and which health professionals are involved.[6] Whilst some centres followed-up patients beyond 10 years, others did not have an established follow-up policy and in some centres follow-up services have been curtailed or stopped entirely

For peer review only

after an early post-operative check.[6] Notably, we do not know whether long-term follow-up is cost-effective or whether disinvestment is safe for patients.

This project aims to determine the consequences of disinvestment in hip and knee arthroplasty follow-up. Given the timeline from joint replacement to revision, with a 7% revision rate for THA and 4% revision rate for TKA at 14 years, conducting a randomised controlled trial to address this question is not feasible. Moreover, the huge variation in follow-up practice across the UK limits the generalisability of the results of an RCT. We will therefore use a mixed-methods approach to comprehensively evaluate the requirements for arthroplasty follow-up and will use this evidence to inform the development of consensus-based recommendations and a policy document which includes a stratification algorithm to determine appropriate follow-up for individual patients. Disinvestment is a complex and often contentious issue. We plan to make use of published recommendations[7] to ensure that the results of this work are understood and considered as a genuine attempt to use the best evidence available to ensure that the NHS gets value for money and that patients remain safe.

For peer review only

Methods and analysis

Study objectives

A. Identify who needs follow-up and when this should occur for primary THA, TKA, and unicompartmental knee arthroplasty (UKA) surgery by making use of routinely-collected NHS data

B. Understand the patient journey (in primary and secondary care) to revision surgery by recruiting patients admitted for elective and emergency hip and knee revision surgery

C. Establish how and when patients are identified for revision, why some patients are missed from regular follow-up and present acutely with fracture around the implant (peri-prosthetic fracture), by using prospective and retrospective data

D. Identify the most appropriate and cost-effective follow-up pathway to minimise potential harm to patients by undertaking cost-effectiveness modelling

E. Provide evidence- and consensus-based recommendations on how follow-up of primary THA and TKA should be conducted.

Design

This is a mixed-methods study using a variety of data sources consisting of four interconnected work packages (WP): 1) a systematic literature review; 2a) analysis of routinely-collected NHS data to understand when and which patient present for revision surgery; 2b) prospective data regarding how patients currently present for revision surgery collected on around 455 patients prior to elective or emergency revision surgery; 3) economic modelling to simulate long-term costs and quality adjusted life years associated with different follow-up models; 4) a Delphi-consensus process, incorporating all previous work packages and involving all stakeholders, to develop a policy document which includes a stratification algorithm to determine appropriate follow-up for an individual patient.

Work package 1: Systematic review

The aim of the review is to evaluate different models of routine long-term follow-up care after TKA/THA/UKA. This systematic review will establish a robust evidence base for the cost-effectiveness modelling (WP3) and consensus guideline development (WP4).

For peer review only - http://bmjopen.bmj.com/site/about/guidelines.xhtml

Registration

This systematic review will be undertaken following Cochrane Collaboration methods[8] and reported in accordance with PRISMA (Preferred Reporting Items for Systematic Review and Meta-analyses) guidelines.[9] It has been prospectively registered with PROSPERO (CRD42017053017).

Searches

A comprehensive literature search will be undertaken with the aim of retrieving all relevant literature, published or unpublished, which evaluated the effectiveness of long-term follow-up after primary TKA/THA/UKA. A range of information sources will be searched: BIOSIS, CINAHL, ClinicalTrials.gov, The Cochrane Library, Embase, Health Management Information Consortium, IDEAS (RePEC), Ovid Medline(R), ProQuest Dissertations & Theses, PsycINFO, PubMed and Web of Science. Reference lists of included studies will be reviewed for potentially relevant articles. A sample search strategy is detailed in appendix A. No date or language restrictions will be applied.

Criteria for selection of studies

All study designs will be included which either i) consider the clinical and/or cost effectiveness of routine long-term (>5 years) follow-up care after primary THA, TKA, or UKA; ii) describe patient safety issues associated with routine follow-up; or iii) consider the acceptability of new care pathways from the perspective of the patient and/or practitioner. Studies will be excluded if they do not report specific patient-related outcome measures or appropriate health utility measures.

Selection of studies

Titles/abstracts of identified studies will be screened for eligibility by one experienced reviewer with a random selection (25%) independently screened by a second. Potential studies will be retrieved in full text and reviewed against the inclusion/exclusion criteria independently by the same two reviewers, with a third reviewer used to settle any disputes.

Data extraction

For peer review only

Data will be extracted by a single reviewer using a standardised pro--forma capturing i) purpose and design; ii) methodological characteristics; iii) information relating to quality assessment; iv) outcome data relating to the clinical and cost-effectiveness of routine long-term follow-up care.

Quality assessment

The Cochrane Risk of Bias assessment tool will be used for experimental studies,[10] and the Newcastle-Ottawa scales for cohort and case-control studies.[11] Qualitative literature will be assessed using critical interpretive synthesis.[12] Economic evaluations will be assessed using the Drummond checklist.[13] Studies will be evaluated independently by two reviewers, with a third to settle any disputes. Studies at high risk of bias will not be excluded and conclusions will incorporate observed biases.

Evidence Synthesis

The design, methodological characteristics, study quality and main findings will be summarised in narrative and tabular form. We anticipate substantial heterogeneity amongst included studies precluding the use of meta-analysis techniques.

Work package 2a: Analysis of routinely collected NHS data

This work package will use routinely-collected NHS data to determine when revision happens and to identify patients most likely to require revision in order to target when and on whom follow-up should occur.

Data sources:

Data from five national datasets will be used: 1) Clinical Practice Research Database (CPRD)[14], 2) ResearchOne (RO),[15] 3) Hospital Episode Statistics (HES),[16] 4) National Joint Registry (NJR),[17] and 5) Patient Reported Outcome Measures (PROMs)[18].

Three linked datasets will be constructed for analysis: (a) CPRD-HES-PROMS, which pre-exists at the University of Oxford, (b) RO-HES will be constructed and analysed at the University of Leeds. Linkage will be undertaken by NHS Digital on the basis of pseudonyms generated from NHS numbers by the data providers (c)

For peer review only

NJR-HES-PROMS will be constructed and analysed at the University of Oxford. Linkages will be undertaken by NHS Digital, using an agreed set of common patient identifiers, including NHS number. Datasets (a) and (b) provide a primary care view (e.g. prior diagnoses, prescribing) and include different, representative patient populations for cross-validation, (c) provides a secondary care view (e.g. surgeon, procedure details).

Data analysis:

The primary outcome of the analysis will be mid-late term revision (>5 years post primary surgery), defined as the removal, exchange or addition of any of the components of arthroplasty. Exposures will include secondary care predictors, including patient level characteristics recorded in NJR and HES (e.g. age, BMI), surgical and operative factors, and symptoms of pain, function and health-related quality of life pre-operatively and six-months post-surgery from PROMS, and primary care predictors, including patient demographics, comorbidities and use of drugs which can affect fracture risk. Survival analysis will be used to model time to revision.[19, 20] The smoothed Nelson-Aalen cumulative hazard rate will be examined to identify any peak in the mid-long term risk of revision. Cox proportional hazards regression modelling will be used to identify pre, peri- and post-operative predictors of mid-late term revision e.g. age, BMI, co-morbidities, implant type, surgeon skill, and post-operative problems. Competing risk regression will be used, since mortality can be regarded as a competing risk for revision surgery.[21, 22] To account for clustering within the data (such as patients nested within hospitals), a multilevel survival model will be fitted by extending the survival regression model to include a frailty term with a Gaussian distribution.[23]

Work Package 2b Part 1: Multicentre observational prospective cohort

Prospective data collection from patients undergoing revision surgery.

Objectives:

- Identify all recent (previous 12 months) medical appointments and advice sessions related to the index joint in primary and secondary care
- Establish if the patient has been seen by orthopaedic health professionals from 12 months after primary surgery until this hospital admission, i.e. was the

For peer review only

revision directed by routine follow-up

Design:

A multi-centre, observational, single visit, prospective cohort study of patients admitted for revision hip or knee surgery.

Population:

Patients presenting for elective and emergency revision surgery of a primary THA, TKA or UKA, and who are able and willing to provide written informed consent will be included in the study. Patients will be excluded if they have had previous revision surgery; metal-on-metal primary joint replacement; or hip hemi-arthroplasty. Participants will be recruited from a sample of hospitals selected to provide geographical spread and representation of teaching hospitals, district general hospitals and hospitals with a special interest in joint replacement

Data collection:

A participant case report form (CRF) will capture details of follow-up after primary surgery and pathway to current revision surgery, including symptom state. An investigator CRF will extract data from medical notes including demographics (age, gender, diagnosis leading to primary surgery, medical history), GP and hospital appointments, details of primary and revision surgery (including implant type, complications, length of stay). The participant CRF will be piloted with the Leeds Biomedical Research Centre Patient and Public Involvement (PPI) group and the investigator CRF with two research nurses to ascertain the comprehension, usability and completeness of data subsequently extracted.

Sample size:

We will use stratified sampling to recruit centres of varying size and anticipate that the average number of patients per centre will be 45 (based on NJR records and information from prospective centres). We initially anticipated the recruitment of 25 centres. With a recruitment rate of 60% this gave 27 recruited patients from 25 centres (N=675). We do not know the intra-class correlation coefficient (ICC) for our primary outcome ("Was the revision a result of routine follow-up?"), but we anticipate it to be in the region of 0.01 to 0.05. To be conservative, we use ICC=0.05. This

For peer review only

gives a design factor of 2.3 and hence an effective sample size of 293 after accounting for clustering within centre. The enrolment of 35 centres reduced the design factor to 1.6 and the total sample size required to 455. From previous research[6] we estimate that the rate of our primary outcome is 20% so that the effective number of events will be 58. Hence we will have sufficient power for our logistic regression to robustly estimate the coefficients of up to five potential risk factors derived from our brief patient survey.[24]

Analysis:

The primary outcome will be ‘revision identified through routine follow-up’, and this will be modelled through a multi-level logistic regression model, with a centre-level random intercept of particular interest. The size of the centre-level effect will be assessed as the proportion of variance explained and will also be assessed through a likelihood ratio test. Up to five factors from the patient questionnaire will be explored as fixed-effects at the patient level. This will adjust for case mix. Factors that are found to be both clinically and statistically significant could potentially contribute to a stratified approach to follow-up.

Work Package 2b Part 2: Qualitative Study

Building on previous work highlighting the changes in follow-up practice,[6] this work-package aims to explore the rationale and motivating factors behind these changes, the facilitators, and the evidence considered when implementing new pathways, including no follow-up.

Sampling:

A sample of n=20-30 orthopaedic practitioners and/or unit managers will be recruited. Purposive sampling via sampling matrix will recruit participants with different experiences of a range of follow-up pathways while reflecting NHS Trust type, geographical area (urban, rural); socio-economic area (low/high socio-economic status); diverse ethnicity. Some selection criteria are likely to be nested (e.g. hospital type, geographical area) and care will be taken to ensure that all viewpoints are represented.

Data collection:

For peer review only

Semi-structured, telephone interviews following a topic guide refined from the literature review and expert opinion (clinician co-applicants/advisors and PPI members). The researcher will probe pertinent initial responses and expand on issues raised. Interviews will be recorded and transcribed verbatim.

Data analysis:

The guiding approach will be Framework Analysis.[25] Data analysis will comprise five stages: i) data familiarisation; ii) identifying the thematic framework; iii) indexing; iv) charting; v) mapping and interpreting. The process of familiarisation enables the researcher to identify emerging themes or issues in the data. Little is known about why NHS Trusts have chosen to either withdraw follow-up care or change the way it is delivered. The evidence generated from the literature review and input from our clinical co-applicants will be used to help identify and refine the thematic framework. Themes are flexible and can be modified in the light of new data, and a process of constant comparison will be undertaken across themes and cases.

Work package 3:

As previous work conducted by members of our team has identified considerable heterogeneity in current follow-up pathways,[6] our cost-effectiveness analysis will compare the relative costs and quality-adjusted life years associated with having follow-up compared to not having follow-up. A third hypothetical scenario of a virtual follow-up will be considered.

Comparators:

Both the findings from our systematic review and the prospective cohort will inform the criteria to be used to identify patients as having or not having follow-up. The seven-year reference point for a follow-up currently suggested by BHS and BOA guidelines is likely to be incorporated. Patients having an orthopaedic outpatient appointment around the reference point(s) following a primary arthroplasty will be used to group patients in the CPRD-HES-PROMS dataset into the follow-up and no-follow-up groups. Joint-specific revision procedures will be identified by OPCS-4 codes as reported in the Admitted Patient Care dataset within HES, with corresponding linked records to primary care and PROMS.

For peer review only

For peer review only - http://bmjopen.bmj.com/site/about/guidelines.xhtml

Model structure:

To identify the most appropriate modelling approach for the question and data at hand, we will conduct a series of preliminary analysis to determine if a cohort-level or patient-level decision analytic model should be employed. Previous models examining the long-term cost-effectiveness of hip and knee replacements have used cohort Markov models.[26, 27] Analyses will include associations between patients' characteristics and revision rates, health utilities and costs, and whether the risk for revision depends on the time patients stay unrevised after their primary. Regardless of the chosen model type, the key health state or event will be revision arthroplasty, with death and complications also considered. The model will be designed to cover patients' lifetime and analysed from an NHS and Personal Social Services perspective, with discounting of costs and outcomes as per current guide to the methods of technology appraisal.[28]

Model inputs:

WP2 datasets will be used to quantify primary and hospital healthcare resource use for comparator groups of follow-up care models through estimation of NHS costs and health-related quality of life (HRQoL). The economic model will simulate long-term costs and quality adjusted life years (QALYs) associated with each care model. Primary care costs will include consultations, and hospital costs will be derived by grouping hospital episodes into Health Resource Groups, a set of casemix groupings utilising similar levels of healthcare resources. Panel data regression analysis[29-31] will be used to estimate hospital costs conditional on patient characteristics and co-morbidities. QALYs and transition probabilities will be derived from the linked datasets and published literature as needed. The hypothetical costs of virtual follow-up will be based on similar virtual clinic alternatives previously studied and NHS x-ray-associated costs.

Analysis:

Cost-effectiveness analyses will be performed separately for relevant patient subgroups based on gender, age and other potential covariates for which data may be available. As with all economic models, a number of assumptions will be made, and their plausibility and potential impact discussed, relating to model structure and input parameters for transition probabilities, health utilities and costs, including the

For peer review only

cost of periprosthetic fractures if no reference is found for these in the literature. Sensitivity analyses will be conducted to explore the uncertainty associated with key assumptions and model parameters, and the implications of using different estimates discussed.

Work package 4: Delphi-consensus process

This work package will use the collective evidence from WP1-3 to inform a consensus process to determine appropriate follow-up care pathways for hip and knee arthroplasty

Evidence gathered from WP1-3 will feed into a consensus panel workshop. We intend to use methods employed by the National Institute for Health and Care Excellence (NICE) in both the technology assessment committees and Guideline Development Groups. The expert stakeholders invited to attend will have a special interest in patient follow-up after hip or knee replacement surgery. Participants will include patients, orthopaedic surgeons, arthroplasty practitioners, NHS managers and commissioners, manufacturers and representatives of the major orthopaedic bodies (including BOA, BHS and BASK). The purpose of this exercise is to consider the evidence and obtain agreement for future care pathways, supported by the evidence of their effectiveness and cost-effectiveness, to be recommended and adopted across the NHS. Following the NICE consensus model all participants will receive summaries of the main research findings in advance. There will be presentations from the work-stream leaders to outline the evidence for consideration.

Robert et al (2014) demonstrate that decommissioning is often about more than the 'evidence' and that withdrawal of previously available services is often seen as being driven by the wrong kind of evidence, based on cost data and political priorities and not on what patients and service users value.[7] It is a complex issue, perhaps as contentious as NICE decisions when they do not fund an effective intervention because it exceeds the threshold. However, NICE investment decisions are made with the explicit understanding that, with no increase in the budget, there must be some displacement of other health care technologies.[32] We plan to make use of the recommendations for engagement and the use of evidence outlined in Robert et al to ensure the results of this work are understood and considered as a genuine

For peer review only - http://bmjopen.bmj.com/site/about/guidelines.xhtml

attempt to use the best evidence available to ensure that the NHS gets value for money and that patients remain safe.

Patient and Public Involvement

Members of the Leeds BRC, Oxford and Bristol PPI groups are involved in UK SAFE. The PPI co-applicant is a member of the study steering committee and contributes across all work packages. Two independent PPI advisors sit on the Independent Advisory Group. Specific areas where lay involvement will be pivotal include the interpretation of results of the systematic review, the expert panel discussion and consensus process, study oversight (steering group), preparation of patient material and study results and contribution to reports and newsletters for patients and NHS staff.

Ethics and dissemination

All studies will be conducted in accordance with the principles of Good Clinical Practice, and the UK Policy Framework for Health and Social Care Research, 2018. Favourable ethical opinion has been obtained for WP2a (RO-HES) (220520) and WP2B (220316) from the National Research Ethics Committee. Following advice from the Confidentiality Advisory Group (17/CAG/0122), data controllers for the datasets used in WP2a (RO-HES) – NHS Digital and The Phoenix Partnership – confirmed that Section 251 support was not required as no identifiable data was flowing into or out of these parties. Application for approval of WP2a (RO-HES) from the Independent Group Advising on the Release of Data (IGARD) at NHS Digital is in progress (DARS-NIC-147997). Section 251 support (17/CAG/0030) and NHS Digital approval (DARS-NIC-172121-G0Z1H-v0.11) have been obtained for WP2a (NJR-HES-PROMS). ISAC (11_050MnA2R2) approval has been obtained for WP2a (CPRD-HES).

At the end of the project, outputs will be disseminated nationally in the form of an executive summary statement of the agreed pathway/s through appropriate NHS Networks, NICE, the NHS England Elective Orthopaedics Sub-committee, the NHS Institute for Innovation and Improvement and professional societies, including BHS, BOA, BASK, Arthroplasty Care Practitioners Association (ACPA) and the NJR. Dissemination will be key to developing a culture of ‘finding the best way of doing

For peer review only

something and doing it everywhere' to significantly reduce wastage of clinical resources and optimise NHS spend. We will put forward the consensus statement to each society's AGM for adoption as a resolution. Internationally, dissemination platforms are in place through the International Society of Arthroplasty Registers (ISAR) and the European Federation of National Associations of Orthopaedics and Traumatology (EFORT). A lay summary of the project will be produced for study participants. Findings will also be presented at relevant orthopaedic and methodological conferences, such as the BOA and the Exploiting Existing Data for Health Research conference. The chief investigator and co-applicants will be named as authors on main publications, and an appropriate first author agreed through discussion. Other key individuals will be included as authors or contributors as appropriate, at the discretion of the Senior Management Group (SMG). Any disputes relating to authorship will be resolved by the Steering Committee.

The Chair and Independent members of the Steering Committee will be acknowledged, but will not qualify for full authorship, in order to maintain their independence. Individual collaborators must not publish data concerning their participants' which are directly relevant to the questions posed in the study until the main results of the study have been published.

Conclusion

This research will deliver the first research-supported, best-for-patient, joint-specific, cost-effective recommendations for follow-up pathways, providing a gold standard for clinical excellence, and follow-up advice for patients, surgeons, purchasers and the NHS as a whole. Value is not limited to the UK, but has substantial global impact potential.

The impact of this work will be to reduce the burden on patients and the NHS in terms of outpatient visits and clinical tests that do not add benefit, while optimising detection of potential problems. From an NHS perspective, this work will provide managers with economic and clinical information on arthroplasty follow-up to inform service planning and delivery, and the role of arthroplasty practitioners in this service, with the potential to reduce geographical disparity through NHS trusts modelling their service provision on a national evidence-based guideline; provide orthopaedic

For peer review only

surgeons with guidance on follow-up, including patient and economic considerations of factors involved; produce arthroplasty follow-up guidelines for adoption by the relevant specialist societies and information for their members. From a patient perspective, this work will help to inform patients about follow-up practice, empower them to make choices about future healthcare relating to their joint arthroplasty and provide reassurance that their follow-up pathway is appropriate

The outputs of this project, in terms of evidence-based support for timing of follow-up and identification of the most cost-effective follow-up model, fit directly within the NHS framework for improving outcomes from elective procedures. Rationalising current diversity of follow-up practices should enable substantial savings for the NHS. We envisage outputs to be readily applicable to the wider NHS, not only hip and knee but also other joint replacements. With the committed support of key national and international organisations already in place, we anticipate that these guidelines will be positively received and that implementation will be widespread.

For peer review only

References

1. National Joint Registry for England, Wales, Northern Ireland and the Isle of Man: 15th Annual report 2018 http://www.njrreports.org.uk/; 2018.

2. Vanhegan IS, Malik AK, Jayakumar P, et al. A financial analysis of revision hip arthroplasty: the economic burden in relation to the national tariff. *J Bone Joint Surg Br* 2012;94(5):619-23.

3. Briggs T. Getting it right first time. Improving the quality of orthopaedic care within the National Health Service in England2012.

4. Singh JA. Epidemiology of Knee and Hip Arthroplasty: A Systematic Review. *The Open Orthopaedics Journal* 2011;5:80-5.

5. British Orthopaedic Association. Primary total hip replacement: a guide to good practice. 2012.

6. Smith LK. A Survey of the Current State of Hip Arthroplasty Surveillance in the United Kingdom. *Musculoskeletal Care* 2014;12(4):232-8.

7. Robert G, Harlock J, Williams I. Implementation Science. 2014;9:123.

8. Higgins JPT GSe. Cochrane Handbook for Systematic Reviews of Interventions Version 5.1.0 [updated March 2011]. The Cochrane Collaboration, 2011. Available from http://handbook.cochrane.org.

9. Liberati A, Altman DG, Tetzlaff J, et al. The PRISMA Statement for Reporting Systematic Reviews and Meta-Analyses of Studies That Evaluate Health Care Interventions: Explanation and Elaboration. *PLOS Medicine* 2009;6(7):e1000100.

10. Higgins JP, Altman DG, Sterne JA. Chapter 8: Assessing risk of bias in included studies in Cochrane handbook for systematic reviews of interventions Version 5.1.0: Available from http://handbook.cochrane.org.; 2011.

11. Wells G, Shea B, O'Connell D, et al. The Newcastle-Ottawa Scale (NOS) for assessing the quality of nonrandomised studies in meta-analyses

http://www.ohri.ca/programs/clinical_epidemiology/oxford.asp; 2014.

12. Dixon-Woods M, Cavers D, Agarwal S, et al. Conducting a critical interpretive synthesis of the literature on access to healthcare by vulnerable groups. *BMC Med Res Methodol* 2006;6(1):35.

13. Drummond MF, Schulpher MJ, Claxton K, et al. Methods for the Economic Evaluation of Health Care Programmes (4th edition). Oxford: Oxford University Press; 2015.

14. http://www.cprd.com

15. http://www.researchone.org

16. http://www.hscic.gov.uk/hes.

17. http://www.njrcentre.org.uk.

18. http://www.hscic.gov.uk/hesproms.

19. Pinedo-Villanueva R, Turner D, Raftery JP, et al. Outcomes after total hip replacement. *Osteoarthritis Cartilage* 2014;22:S214.

For peer review only

20. Pinedo-Villanueva R, Turner D, Raftery JP, et al. Primary care costs attributable to osteoarthritis before total hip replacement. *Osteoarthritis and Cartilage* 2014;22:S210-S1.

21. Jason PF, Gray RJ. A proportional hazards model for the subdistribution of a competing risk. *Journal of the American Statistical Association* 1999;94(446):496-509.

22. Prieto-Alhambra D, Pages-Castella A, Wallace G, et al. Predictors of fracture while on treatment with oral bisphosphonates: a population-based cohort study. *J Bone Miner Res* 2014;29(1):268-74.

23. Hirsch K, Wienke A. Software for semiparametric shared gamma and log-normal frailty models: An overview. *Comput Methods Programs Biomed* 2012;107(3):582-97.

24. Peduzzi P, Concato J, Kemper E, et al. A simulation study of the number of events per variable in logistic regression analysis. *J Clin Epidemiol* 1996;49(12):1373-9.

25. Wallace G, Judge A, Prieto-Alhambra D, et al. The effect of body mass index on the risk of post-operative complications during the 6 months following total hip replacement or total knee replacement surgery. *Osteoarthritis Cartilage* 2014;22(7):918-27.

26. Burn E, Liddle AD, Hamilton TW, et al. Cost-effectiveness of unicompartmental compared with total knee replacement: a population-based study using data from the National Joint Registry for England and Wales. *BMJ Open* 2018;8(4):e020977.

27. Fitzpatrick R, Shortall E, Sculpher M, et al. Primary total hip replacement surgery: a systematic review of outcomes and modelling of cost-effectiveness associated with different prostheses. *Health Technol Assess* 1998;2(20):1-64.

28. https://www.nice.org.uk/process/pmg9/chapter/foreword.

29. Basu A, Rathouz PJ. Estimating marginal and incremental effects on health outcomes using flexible link and variance function models. *Biostatistics* 2005;6(1):93-109.

30. Clarke P, Leal J, Kelman C, et al. Estimating the cost of complications of diabetes in Australia using administrative health-care data. *Value Health* 2008;11(2):199-206.

31. Mullahy J. Much ado about two: reconsidering retransformation and the two-part model in health econometrics. *J Health Econ* 1998;17(3):247-81.

32. https://www.nice.org.uk/about/what-we-do/our-programmes/nice-guidance/nice-technology-appraisal-guidance.

For peer review only

Authors' contributions

CCM - contributed to the planning and design of this study, drafted the article, revised the article and approved the final manuscript.

SRK - contributed to the planning and design of this study, drafted the article, revised the article and approved the final manuscript.

NA - contributed to the planning and design of this study, revised the article and approved the final manuscript

JH - contributed to the planning and design of this study, revised the article and approved the final manuscript

AJ - contributed to the planning and design of this study, revised the article and approved the final manuscript

JM - contributed to the planning and design of this study, revised the article and approved the final manuscript

JO - contributed to the planning and design of this study, revised the article and approved the final manuscript

RPV - contributed to the planning and design of this study, revised the article and approved the final manuscript

LKS - contributed to the planning and design of this study, revised the article and approved the final manuscript

CS - contributed to the planning and design of this study, revised the article and approved the final manuscript

CMT - contributed to the planning and design of this study, revised the article and approved the final manuscript

RW - contributed to the planning and design of this study, revised the article and approved the final manuscript

JMW - contributed to the planning and design of this study, revised the article and approved the final manuscript

PGC - contributed to the planning and design of this study, revised the article and approved the final manuscript

MS - contributed to the planning and design of this study, revised the article and approved the final manuscript

Funding statement

For peer review only

This article presents independent research funded by the National Institute for Health Research (NIHR) Health Services and Delivery Research Programme (14/70/146) and by the NIHR Leeds Biomedical Research Centre (BRC).

Disclaimer

The views expressed are those of the author(s) and not necessarily those of the NIHR or the Department of Health and Social Care.

Competing interests

None declared.

For peer review only

Appendix 1
Ovid MEDLINE(R) Epub Ahead of Print, In-Process & Other Non-Indexed Citations, Ovid MEDLINE(R) Daily and Ovid MEDLINE(R) 1946 to Present
Search Strategy:
----
1 Arthroplasty, Replacement, Knee/ (18061)
2 Knee Prosthesis/ (10316)
3 (TKA or TKR or UKR).tw. (9131)
4 Arthroplasty, Replacement, Hip/ (21968)
5 Hip Prosthesis/ (21263)
6 (THA or THR).tw. (30477)
7 or/1-6 (79707)
8 Hip/ (11074)
9 Osteoarthritis, Hip/ (7521)
10 Hip Joint/ (24959)
11 Hip?.tw. (121242)
12 Femur Head/ (8758)
13 Acetabulum/ (9886)
14 ("Femur head*" or "femoral head*" or acetabul*).tw. (27939)
15 "Total joint".tw. (4508)
16 Knee/ (12862)
17 Knee Joint/ (47967)
18 Osteoarthritis, Knee/ (15086)
19 Knee?.tw. (122420)
20 or/8-19 [Knee or Hip joints] (259908)
21 Joint Prosthesis/ (9772)
22 "Prostheses and Implants"/ (43103)
23 (Arthroplast* or replace* or implant* or prosthes* or unicompartment*).tw. (724712)
24 (Surf* or resurf*).tw. (990965)
25 or/21-24 [Arthroplasty] (1680546)
26 and/20,25 (82595)
27 7 or 26 [Hip or Knee Arthoplasty] (113853)
28 Longitudinal studies/ (109550)
29 Prospective studies/ (457925)
30 Time/ or time factors/ (1126356)
31 Follow-up studies/ (586823)
32 Epidemiological Monitoring/ (5660)
33 or/28-32 [Follow-up Studies MeSH] (2051292)
34 Retreatment/ (7424)
35 Reoperation/ (76777)
36 Treatment failure/ (31517)
37 exp Postoperative Complications/ (482686)
38 exp Prosthesis failure/ (25670)
39 or/34-38 [Complications MESH] (555690)
40 Risk factors/ (699996)
41 33 or 39 or 40 [Long term complications or risks MESH] (2963609)
42 *Postoperative Care/ (14831)
43 Postoperative care/mt (9905)
44 Postoperative Period/ (42528)
45 Aftercare/ (7484)
46 or/42-45 [Post Operative Care MeSH] (69319)
47 41 and 46 [Post op follow up MeSH] (29208)
48 Critical Pathways/ (5783)
49 47 or 48 [Post op follow up or pathways MeSH] (34925)

For peer review only

50 (("After care" or aftercare or "after surgery" or "after arthroplas*") adj3 follow-up).tw. (2471)
51 ((Postoperati* or post-operati* or "post surger*" or "post arthroplast*") adj3 follow-up).tw. (13670)
52 ((pathway* or care or treatment* or appointment* or consultation*) adj3 follow-up).tw. (29535)
53 ((Postoperati* or post-operati* or "post surger*" or "post arthroplast*") adj3 (surveillance* or monitor*)).tw. (4101)
54 or/50-53 [Follow-up studies Textword] (49052)
55 (("After care" or aftercare or "after surgery" or "after arthroplas*") adj3 (failur* or reoperat* or re-operat* or readmission or readmit* or revision or revisions)).tw. (691)
56 ((Postoperati* or post-operati* or "post surger*" or "post arthroplast*") adj3 (failure* or reoperat* or re-operat* or readmission or readmit* or revision or revisions)).tw. (5650)
57 ((pathway* or care or treatment* or appointment* or consultation* or follow-up or time or risk*) adj8 (revis* adj2 surgery)).tw. (1505)
58 or/55-57 [Post op complications Textword] (7776)
59 (("After care" or aftercare or "after surgery" or "after arthroplas*") adj3 risk*).tw. (637)
60 ((Postoperati* or post-operati* or "post surger*" or "post arthroplast*") adj3 risk).tw. (12221)
61 or/59-60 [Post op risks Textword] (12801)
62 (("After care" or aftercare or "after surgery" or "after arthroplas*") adj3 pathway*).tw. (93)
63 ((Postoperati* or post-operati* or "post surger*" or "post arthroplast*") adj3 pathway*).tw. (181)
64 (Care pathway* or clinical pathway* or critical pathway*).tw. (6772)
65 or/62-64 [Post op pathways Textword] (6956)
66 54 or 58 or 61 or 65 [Post op follow up Textword] (75424)
67 49 or 66 [Post Op Follow Up] (106039)
68 27 and 67 [TJA Post op follow up] (3634)

For peer review only

BMJ Open

Towards UK poSt Arthroplasty Follow-up rEcommendations (UK SAFE): protocol for an evaluation of the requirements for arthroplasty follow-up, and the production of consensus-based recommendations

Journal:	*BMJ Open*
Manuscript ID	bmjopen-2019-031351.R1
Article Type:	Protocol
Date Submitted by the Author:	21-May-2019
Complete List of Authors:	Czoski Murray, Carolyn; University of Leeds Leeds Institute of Health Sciences<br>Kingsbury, Sarah; University of Leeds Leeds Institute of Rheumatic and Musculoskeletal Medicine, Leeds Institute of Rheumatic and Musculoskeletal Medicine; NIHR Leeds Biomedical Research Centre,<br>Arden, Nigel; University of Oxford Nuffield Department of Orthopaedics Rheumatology and Musculoskeletal Sciences<br>Hewison, Jenny; University of Leeds Leeds Institute of Health Sciences<br>Judge, Andrew; University of Bristol, Musculoskeletal Research Unit, Translational Health Sciences, Bristol Medical School, University of Bristol, Learning and Research Building, Level 1, Southmead Hospital, Southmead, BS10 5NB; MRC Lifecourse Epidemiology Unit<br>Matu, Jamie; University of Leeds Leeds Institute of Health Sciences; University of Leeds Leeds Institute of Rheumatic and Musculoskeletal Medicine<br>O'Shea, Jamie; Manor Park Surgery<br>Pinedo-Villanueva, Rafael; University of Oxford Nuffield Department of Orthopaedics Rheumatology and Musculoskeletal Sciences, Nuffield Department of Orthopaedics Rheumatology and Musculoskeletal Sciences<br>Smith, Lindsay; Weston Area Health NHS Trust, Trauma and Orthopaedics; Weston Area Health NHS Trust<br>Smith, Chris; University of Leeds Leeds Institute of Health Sciences<br>Thomas, Christine; NIHR Leeds Biomedical Research Centre; University of Leeds Leeds Institute of Rheumatic and Musculoskeletal Medicine<br>West, Robert; University of Leeds Leeds Institute of Health Sciences<br>Wright, Judy; University of Leeds Leeds Institute of Health Sciences<br>Conaghan, Philip; University of Leeds, Leeds Institute of Rheumatic and Musculoskeletal Medicine and NIHR Biomedical Research Centre<br>Stone, Martin; NIHR Leeds Biomedical Research Centre; Leeds Teaching Hospitals NHS Trust
**Primary Subject Heading**:	Health services research
Secondary Subject Heading:	Surgery
Keywords:	Hip < ORTHOPAEDIC & TRAUMA SURGERY, Knee < ORTHOPAEDIC & TRAUMA SURGERY, Organisation of health services < HEALTH SERVICES

	ADMINISTRATION & MANAGEMENT, Quality in health care < HEALTH SERVICES ADMINISTRATION & MANAGEMENT, Risk management < HEALTH SERVICES ADMINISTRATION & MANAGEMENT

Title: Towards UK poSt Arthroplasty Follow-up rEcommendations (UK SAFE): protocol for an evaluation of the requirements for arthroplasty follow-up, and the production of consensus-based recommendations

Authors names:

Carolyn Czoski Murray[1*], Sarah R Kingsbury[2,3*], Nigel K Arden[4,5], Jenny Hewison[1], Andrew Judge[5,6,7], Jamie Matu[1,2], Jamie O'Shea[8], Rafael Pinedo-Villanueva[4], Lindsay K Smith[9], Chris Smith[1], Christine M Thomas[3], Robert West[1], Judy M Wright[1], Philip G Conaghan[2,3^], Martin Stone[3,10^],

* These authors contributed equally to this work

^ These authors contributed equally to this work

Department and institution:

1 Leeds Institute of Health Sciences, University of Leeds, Leeds, UK

2 Leeds Institute of Rheumatic and Musculoskeletal Medicine, University of Leeds, Leeds, UK

3 NIHR Leeds Biomedical Research Centre, Leeds, UK

4 Nuffield Department of Orthopaedics, Rheumatology and Orthopaedic Sciences, University of Oxford, Oxford, UK.

5 MRC Lifecourse Epidemiology Unit, University of Southampton, Southampton.

6 Pharmaco- and Device-Epidemiology Group, Centre for Statistics in Medicine, NDORMS, University of Oxford, Oxford.

7 Translational Health Sciences, University of Bristol, Bristol.

8 Manor Park Surgery, Leeds, UK

9 Faculty of Health & Applied Sciences, University of the West of England, Glenside Campus, Bristol, UK.

10 Leeds Teaching Hospitals NHS Trust, Leeds UK

For peer review only

Corresponding author:

Philip G Conaghan

Leeds Institute of Rheumatic and Musculoskeletal Medicine,

Chapel Allerton Hospital,

Chapeltown Rd,

Leeds LS7 4SA, UK

Phone: +44 113 3924884

Fax: +44 113 3924991

Email: p.conaghan@leeds.ac.uk

Keywords: joint replacement, hip arthroplasty, knee arthroplasty, orthopaedic surgery, surveillance

Dates of study: 01/12/2016-30/11/2019

Word count: 4091

For peer review only

Abstract

Introduction:

Hip and knee arthroplasties have revolutionised the management of degenerative joint diseases and, due to an ageing population, are becoming increasingly common. Follow-up of joint prostheses is to identify problems in symptomatic or asymptomatic patients due to infection, osteolysis, bone loss or potential peri-prosthetic fracture, enabling timely intervention to prevent catastrophic failure at a later date. Early revision is usually more straight-forward surgically and less traumatic for the patient. However, routine long-term follow-up is costly and requires considerable clinical time. Therefore, some centres in the UK have curtailed this aspect of primary hip and knee arthroplasty services, doing so without an evidence-base that such disinvestment is clinically- or cost-effective.

Methods:

Given the timeline from joint replacement to revision, conducting a randomised controlled trial (RCT) to determine potential consequences of disinvestment in hip and knee arthroplasty follow-up is not feasible. Furthermore the low revision rates of modern prostheses, less than 10% at 10 years, would necessitate thousands of patients to adequately power such a study. The huge variation in follow-up practice across the UK also limits the generalisability of an RCT. This study will therefore use a mixed-methods approach to examine the requirements for arthroplasty follow-up and produce evidence- and consensus-based recommendations as to how, when and on whom follow-up should be conducted. Four interconnected work packages will be completed: 1) a systematic literature review; 2a) analysis of routinely-collected NHS data from five national datasets to understand when and which patients present for revision surgery; 2b) prospective data regarding how patients currently present for revision surgery; 3) economic modelling to simulate long-term costs and quality-adjusted life years associated with different follow-up care models; 4) a Delphi-consensus process, involving all stakeholders, to develop a policy document which includes a stratification algorithm to determine appropriate follow-up care for an individual patient.

For peer review only

Article Summary

Strengths and limitations of this study

- Our mixed-methods approach allows us to address a question that would not be feasible to answer with a randomised controlled trial.

- Our study will capture data from a mixture of teaching hospitals, district general hospitals and hospitals with a special interest in joint replacement and with a geographical spread, increasing the generalisability of our results.

- Our economic model will be populated with routinely-collected NHS data of patients attending primary and hospital care in the UK, ensuring that our analysis is based upon actual patient use of services, outcomes such as health-related quality of life, and costs to the NHS.

- Whilst our analysis is based on data sources that reflect clinical practice in England only, we believe key cost-effectiveness findings are likely to be informative for decision-making in the whole of the UK.

For peer review only

Introduction

Arguably, total hip arthroplasty (THA) and total knee arthroplasty (TKA) are the most successful surgical interventions performed in modern times. Due to an ageing population, and an obesity epidemic, hip and knee replacement procedures increase annually, rising from less than 20,000/year in the UK in 1978 to around 200,000 /year in 2017.[1] The current follow-up requirements are estimated at 500,000–1,000,000 annual outpatient attendances. With limitless resources every patient undergoing a joint arthroplasty would incur routine lifetime follow-up. The rationale for follow-up is to ensure timely detection of complications or arthroplasty failure, such as aseptic loosening, osteolysis, and potential peri-prosthetic fracture. The cost of revision for aseptic loosening is 35% lower than that for peri-prosthetic fractures, and has a lower incidence of complications which impact recovery.[2] However, whilst routine long-term follow-up of joint prostheses may support timely revision for patients with asymptomatic complications, improving long-term health outcomes, it is also costly both clinically and financially.

Orthopaedic services are already one of the poorest performers across the National Health Service (NHS) by failing to meet waiting list targets, with an estimated 8,000 orthopaedic NHS breaches each month.[3] With a rapidly aging population and medical advances that mean less stringent criteria for surgery eligibility,[4] there is no sign that demand will recede in coming years and orthopaedic services will soon reach breaking point. To reduce the burden on orthopaedic services, evidence-based consensus guidelines are required to establish how, when and on whom follow-up should be conducted.

British Hip Society (BHS) and British Orthopaedic Association (BOA) guidelines recommend outpatient follow-up at 1 and 7 years, and every 3 years thereafter for Orthopaedic Data Evaluation Panel 10A (ODEP-10A) implants, with more frequent follow-up for novel implants.[5] However, recent work revealed considerable diversity across the UK in arthroplasty follow-up pathways, in timing, how follow-up is conducted and which health professionals are involved.[6] Whilst some centres followed-up patients beyond 10 years, others did not have an established follow-up policy and in some centres follow-up services have been curtailed or stopped entirely

For peer review only

after an early post-operative check.[6] Notably, we do not know whether long-term follow-up is cost-effective or whether disinvestment is safe for patients.

This project aims to determine the consequences of disinvestment in hip and knee arthroplasty follow-up. Given the timeline from joint replacement to revision, with a 7% revision rate for THA and 4% revision rate for TKA at 14 years, conducting a randomised controlled trial to address this question is not feasible. Moreover, the huge variation in follow-up practice across the UK limits the generalisability of the results of an RCT. We will therefore use a mixed-methods approach to comprehensively evaluate the requirements for arthroplasty follow-up and will use this evidence to inform the development of consensus-based recommendations and a policy document which includes a stratification algorithm to determine appropriate follow-up for individual patients. Disinvestment is a complex and often contentious issue. We plan to make use of published recommendations[7] to ensure that the results of this work are understood and considered as a genuine attempt to use the best evidence available to ensure that the NHS gets value for money and that patients remain safe.

For peer review only

Methods and analysis

Study objectives

A. Identify who needs follow-up and when this should occur for primary THA, TKA, and unicompartmental knee arthroplasty (UKA) surgery by making use of routinely-collected NHS data
B. Understand the patient journey (in primary and secondary care) to revision surgery by recruiting patients admitted for elective and emergency hip and knee revision surgery
C. Establish how and when patients are identified for revision, why some patients are missed from regular follow-up and present acutely with fracture around the implant (peri-prosthetic fracture), by using prospective and retrospective data
D. Identify the most appropriate and cost-effective follow-up pathway to minimise potential harm to patients by undertaking cost-effectiveness modelling
E. Provide evidence- and consensus-based recommendations on how follow-up of primary THA and TKA should be conducted.

Design

This is a mixed-methods study using a variety of data sources consisting of four interconnected work packages (WP): 1) a systematic literature review; 2a) analysis of routinely-collected NHS data to understand when and which patient present for revision surgery; 2b) prospective data regarding how patients currently present for revision surgery collected on around 455 patients prior to elective or emergency revision surgery; 3) economic modelling to simulate long-term costs and quality adjusted life years associated with different follow-up models; 4) a Delphi-consensus process, incorporating all previous work packages and involving all stakeholders, to develop a policy document which includes a stratification algorithm to determine appropriate follow-up for an individual patient.

Work package 1: Systematic review

The aim of the review is to evaluate different models of routine long-term follow-up care after TKA/THA/UKA. This systematic review will establish a robust evidence base for the cost-effectiveness modelling (WP3) and consensus guideline development (WP4).

For peer review only

Registration

This systematic review will be undertaken following Cochrane Collaboration methods[8] and reported in accordance with PRISMA (Preferred Reporting Items for Systematic Review and Meta-analyses) guidelines.[9] It has been prospectively registered with PROSPERO (CRD42017053017).

Searches

A comprehensive literature search will be undertaken with the aim of retrieving all relevant literature, published or unpublished, which evaluated the effectiveness of long-term follow-up after primary TKA/THA/UKA. A range of information sources will be searched: BIOSIS, CINAHL, ClinicalTrials.gov, The Cochrane Library, Embase, Health Management Information Consortium, IDEAS (RePEC), Ovid Medline(R), ProQuest Dissertations & Theses, PsycINFO, PubMed and Web of Science. Reference lists of included studies will be reviewed for potentially relevant articles. A sample search strategy is detailed in appendix A. No date or language restrictions will be applied.

Criteria for selection of studies

All study designs will be included which either i) consider the clinical and/or cost effectiveness of routine long-term (>5 years) follow-up care after primary THA, TKA, or UKA; ii) describe patient safety issues associated with routine follow-up; or iii) consider the acceptability of new care pathways from the perspective of the patient and/or practitioner. Studies will be excluded if they do not report specific patient-related outcome measures or appropriate health utility measures.

Selection of studies

Titles/abstracts of identified studies will be screened for eligibility by one experienced reviewer with a random selection (25%) independently screened by a second. Potential studies will be retrieved in full text and reviewed against the inclusion/exclusion criteria independently by the same two reviewers, with a third reviewer used to settle any disputes.

Data extraction

For peer review only

Data will be extracted by a single reviewer using a standardised pro--forma capturing i) purpose and design; ii) methodological characteristics; iii) information relating to quality assessment; iv) outcome data relating to the clinical and cost-effectiveness of routine long-term follow-up care.

Quality assessment

The Cochrane Risk of Bias assessment tool will be used for experimental studies,[10] and the Newcastle-Ottawa scales for cohort and case-control studies.[11] Qualitative literature will be assessed using critical interpretive synthesis.[12] Economic evaluations will be assessed using the Drummond checklist.[13] Studies will be evaluated independently by two reviewers, with a third to settle any disputes. Studies at high risk of bias will not be excluded and conclusions will incorporate observed biases.

Evidence Synthesis

The design, methodological characteristics, study quality and main findings will be summarised in narrative and tabular form. We anticipate substantial heterogeneity amongst included studies precluding the use of meta-analysis techniques.

Work package 2a: Analysis of routinely collected NHS data

This work package will use routinely-collected NHS data to determine when revision happens and to identify patients most likely to require revision in order to target when and on whom follow-up should occur.

Data sources:

Data from five national datasets will be used: 1) Clinical Practice Research Database (CPRD)[14], 2) ResearchOne (RO),[15] 3) Hospital Episode Statistics (HES),[16] 4) National Joint Registry (NJR),[17] and 5) Patient Reported Outcome Measures (PROMs)[18].

Three linked datasets will be constructed for analysis: (a) CPRD-HES-PROMS, which pre-exists at the University of Oxford, (b) RO-HES will be constructed and analysed at the University of Leeds. Linkage will be undertaken by NHS Digital on the basis of pseudonyms generated from NHS numbers by the data providers (c) NJR-HES-

For peer review only

PROMS will be constructed and analysed at the University of Oxford. Linkages will be undertaken by NHS Digital, using an agreed set of common patient identifiers, including NHS number. Datasets (a) and (b) provide a primary care view (e.g. prior diagnoses, prescribing) and include different, representative patient populations for cross-validation, (c) provides a secondary care view (e.g. surgeon, procedure details).

Data analysis:

The primary outcome of the analysis will be mid-late term revision (>5 years post primary surgery), defined as the removal, exchange or addition of any of the components of arthroplasty. Exposures will include secondary care predictors, including patient level characteristics recorded in NJR and HES (e.g. age, BMI), surgical and operative factors, and symptoms of pain, function and health-related quality of life pre-operatively and six-months post-surgery from PROMS, and primary care predictors, including patient demographics, comorbidities and use of drugs which can affect fracture risk. Survival analysis will be used to model time to revision.[19, 20] The smoothed Nelson-Aalen cumulative hazard rate will be examined to identify any peak in the mid-long term risk of revision. Cox proportional hazards regression modelling will be used to identify pre, peri- and post-operative predictors of mid-late term revision e.g. age, BMI, co-morbidities, implant type, surgeon skill, and post-operative problems. Competing risk regression will be used, since mortality can be regarded as a competing risk for revision surgery.[21, 22] To account for clustering within the data (such as patients nested within hospitals), a multilevel survival model will be fitted by extending the survival regression model to include a frailty term with a Gaussian distribution.[23]

Work Package 2b Part 1: Multicentre observational prospective cohort

Prospective data collection from patients undergoing revision surgery.

Objectives:

- Identify all recent (previous 12 months) medical appointments and advice sessions related to the index joint in primary and secondary care
- Establish if the patient has been seen by orthopaedic health professionals from 12 months after primary surgery until this hospital admission, i.e. was the revision directed by routine follow-up

For peer review only

Design:

A multi-centre, observational, single visit, prospective cohort study of patients admitted for revision hip or knee surgery.

Population:

Patients presenting for elective and emergency revision surgery of a primary THA, TKA or UKA, and who are able and willing to provide written informed consent will be included in the study. Patients will be excluded if they have had previous revision surgery; metal-on-metal primary joint replacement; or hip hemi-arthroplasty. Participants will be recruited from a sample of hospitals selected to provide geographical spread and representation of teaching hospitals, district general hospitals and hospitals with a special interest in joint replacement

Data collection:

A participant case report form (CRF) will capture details of follow-up after primary surgery and pathway to current revision surgery, including symptom state. An investigator CRF will extract data from medical notes including demographics (age, gender, diagnosis leading to primary surgery, medical history), GP and hospital appointments, details of primary and revision surgery (including implant type, complications, length of stay). The participant CRF will be piloted with the Leeds Biomedical Research Centre Patient and Public Involvement (PPI) group and the investigator CRF with two research nurses to ascertain the comprehension, usability and completeness of data subsequently extracted.

Sample size:

We will use stratified sampling to recruit centres of varying size and anticipate that the average number of patients per centre will be 45 (based on NJR records and information from prospective centres). We initially anticipated the recruitment of 25 centres. With a recruitment rate of 60% this gave 27 recruited patients from 25 centres (N=675). We do not know the intra-class correlation coefficient (ICC) for our primary outcome (“Was the revision a result of routine follow-up?”), but we anticipate it to be in the region of 0.01 to 0.05. To be conservative, we use ICC=0.05. This gives a design factor of 2.3 and hence an effective sample size of 293 after

For peer review only

accounting for clustering within centre. The enrolment of 35 centres reduced the design factor to 1.6 and the total sample size required to 455. From previous research[6] we estimate that the rate of our primary outcome is 20% so that the effective number of events will be 58. Hence we will have sufficient power for our logistic regression to robustly estimate the coefficients of up to five potential risk factors derived from our brief patient survey.[24]

Analysis:

The primary outcome will be ‘revision identified through routine follow-up’, and this will be modelled through a multi-level logistic regression model, with a centre-level random intercept of particular interest. The size of the centre-level effect will be assessed as the proportion of variance explained and will also be assessed through a likelihood ratio test. Up to five factors from the patient questionnaire will be explored as fixed-effects at the patient level. This will adjust for case mix. Factors that are found to be both clinically and statistically significant could potentially contribute to a stratified approach to follow-up.

Work Package 2b Part 2: Qualitative Study

Building on previous work highlighting the changes in follow-up practice,[6] this work-package aims to explore the rationale and motivating factors behind these changes, the facilitators, and the evidence considered when implementing new pathways, including no follow-up.

Sampling:

A sample of n=20-30 orthopaedic practitioners and/or unit managers will be recruited. Purposive sampling via sampling matrix will recruit participants with different experiences of a range of follow-up pathways while reflecting NHS Trust type, geographical area (urban, rural); socio-economic area (low/high socio-economic status); diverse ethnicity. Some selection criteria are likely to be nested (e.g. hospital type, geographical area) and care will be taken to ensure that all viewpoints are represented.

Data collection:

For peer review only

Semi-structured, telephone interviews following a topic guide refined from the literature review and expert opinion (clinician co-applicants/advisors and PPI members). The researcher will probe pertinent initial responses and expand on issues raised. Interviews will be recorded and transcribed verbatim.

Data analysis:

The guiding approach will be Framework Analysis.[25] Data analysis will comprise five stages: i) data familiarisation; ii) identifying the thematic framework; iii) indexing; iv) charting; v) mapping and interpreting. The process of familiarisation enables the researcher to identify emerging themes or issues in the data. Little is known about why NHS Trusts have chosen to either withdraw follow-up care or change the way it is delivered. The evidence generated from the literature review and input from our clinical co-applicants will be used to help identify and refine the thematic framework. Themes are flexible and can be modified in the light of new data, and a process of constant comparison will be undertaken across themes and cases.

Work package 3:

As previous work conducted by members of our team has identified considerable heterogeneity in current follow-up pathways,[6] our cost-effectiveness analysis will compare the relative costs and quality-adjusted life years associated with having follow-up compared to not having follow-up. A third hypothetical scenario of a virtual follow-up will be considered.

Comparators:

Both the findings from our systematic review and the prospective cohort will inform the criteria to be used to identify patients as having or not having follow-up. The seven-year reference point for a follow-up currently suggested by BHS and BOA guidelines is likely to be incorporated. Patients having an orthopaedic outpatient appointment around the reference point(s) following a primary arthroplasty will be used to group patients in the CPRD-HES-PROMS dataset into the follow-up and no-follow-up groups. Joint-specific revision procedures will be identified by OPCS-4 codes as reported in the Admitted Patient Care dataset within HES, with corresponding linked records to primary care and PROMS.

For peer review only

Model structure:

To identify the most appropriate modelling approach for the question and data at hand, we will conduct a series of preliminary analysis to determine if a cohort-level or patient-level decision analytic model should be employed. Previous models examining the long-term cost-effectiveness of hip and knee replacements have used cohort Markov models.[26, 27] Analyses will include associations between patients' characteristics and revision rates, health utilities and costs, and whether the risk for revision depends on the time patients stay unrevised after their primary. Regardless of the chosen model type, the key health state or event will be revision arthroplasty, with death and complications also considered. The model will be designed to cover patients' lifetime and analysed from an NHS and Personal Social Services perspective, with discounting of costs and outcomes as per current guide to the methods of technology appraisal.[28]

Model inputs:

WP2 datasets will be used to quantify primary and hospital healthcare resource use for comparator groups of follow-up care models through estimation of NHS costs and health-related quality of life (HRQoL). The economic model will simulate long-term costs and quality adjusted life years (QALYs) associated with each care model. Primary care costs will include consultations, and hospital costs will be derived by grouping hospital episodes into Health Resource Groups, a set of casemix groupings utilising similar levels of healthcare resources. Panel data regression analysis[29-31] will be used to estimate hospital costs conditional on patient characteristics and co-morbidities. QALYs and transition probabilities will be derived from the linked datasets and published literature as needed. The hypothetical costs of virtual follow-up will be based on similar virtual clinic alternatives previously studied and NHS x-ray-associated costs.

Analysis:

Cost-effectiveness analyses will be performed separately for relevant patient subgroups based on gender, age and other potential covariates for which data may be available. As with all economic models, a number of assumptions will be made, and their plausibility and potential impact discussed, relating to model structure and input parameters for transition probabilities, health utilities and costs, including the cost

For peer review only

of periprosthetic fractures if no reference is found for these in the literature. Sensitivity analyses will be conducted to explore the uncertainty associated with key assumptions and model parameters, and the implications of using different estimates discussed.

Work package 4: Delphi-consensus process

This work package will use the collective evidence from WP1-3 to inform a consensus process to determine appropriate follow-up care pathways for hip and knee arthroplasty

Evidence gathered from WP1-3 will feed into a consensus panel workshop. We intend to use methods employed by the National Institute for Health and Care Excellence (NICE) in both the technology assessment committees and Guideline Development Groups. The expert stakeholders invited to attend will have a special interest in patient follow-up after hip or knee replacement surgery. Participants will include patients, orthopaedic surgeons, arthroplasty practitioners, NHS managers and commissioners, manufacturers and representatives of the major orthopaedic bodies (including BOA, BHS and BASK). The purpose of this exercise is to consider the evidence and obtain agreement for future care pathways, supported by the evidence of their effectiveness and cost-effectiveness, to be recommended and adopted across the NHS. Following the NICE consensus model all participants will receive summaries of the main research findings in advance. There will be presentations from the work-stream leaders to outline the evidence for consideration.

Robert et al (2014) demonstrate that decommissioning is often about more than the ‘evidence’ and that withdrawal of previously available services is often seen as being driven by the wrong kind of evidence, based on cost data and political priorities and not on what patients and service users value.[7] It is a complex issue, perhaps as contentious as NICE decisions when they do not fund an effective intervention because it exceeds the threshold. However, NICE investment decisions are made with the explicit understanding that, with no increase in the budget, there must be some displacement of other health care technologies.[32] We plan to make use of the recommendations for engagement and the use of evidence outlined in Robert et al to ensure the results of this work are understood and considered as a genuine attempt to use the best evidence available to ensure that the NHS gets value for money and

For peer review only

that patients remain safe.

Patient and Public Involvement

Members of the Leeds BRC, Oxford and Bristol PPI groups are involved in UK SAFE. The PPI co-applicant is a member of the study steering committee and contributes across all work packages. Two independent PPI advisors sit on the Independent Advisory Group. Specific areas where lay involvement will be pivotal include the interpretation of results of the systematic review, the expert panel discussion and consensus process, study oversight (steering group), preparation of patient material and study results and contribution to reports and newsletters for patients and NHS staff.

Ethics and dissemination

All studies will be conducted in accordance with the principles of Good Clinical Practice, and the UK Policy Framework for Health and Social Care Research, 2018. Favourable ethical opinion has been obtained for WP2a (RO-HES) (220520) and WP2B (220316) from the National Research Ethics Committee. Following advice from the Confidentiality Advisory Group (17/CAG/0122), data controllers for the datasets used in WP2a (RO-HES) – NHS Digital and The Phoenix Partnership – confirmed that Section 251 support was not required as no identifiable data was flowing into or out of these parties. Application for approval of WP2a (RO-HES) from the Independent Group Advising on the Release of Data (IGARD) at NHS Digital is in progress (DARS-NIC-147997). Section 251 support (17/CAG/0030) and NHS Digital approval (DARS-NIC-172121-G0Z1H-v0.11) have been obtained for WP2a (NJR-HES-PROMS). ISAC (11_050MnA2R2) approval has been obtained for WP2a (CPRD-HES).

At the end of the project, outputs will be disseminated nationally in the form of an executive summary statement of the agreed pathway/s through appropriate NHS Networks, NICE, the NHS England Elective Orthopaedics Sub-committee, the NHS Institute for Innovation and Improvement and professional societies, including BHS, BOA, BASK, Arthroplasty Care Practitioners Association (ACPA) and the NJR. Dissemination will be key to developing a culture of ‘finding the best way of doing something and doing it everywhere’ to significantly reduce wastage of clinical resources and optimise NHS spend. We will put forward the consensus statement to

For peer review only

each society's AGM for adoption as a resolution. Internationally, dissemination platforms are in place through the International Society of Arthroplasty Registers (ISAR) and the European Federation of National Associations of Orthopaedics and Traumatology (EFORT). A lay summary of the project will be produced for study participants. Findings will also be presented at relevant orthopaedic and methodological conferences, such as the BOA and the Exploiting Existing Data for Health Research conference. The chief investigator and co-applicants will be named as authors on main publications, and an appropriate first author agreed through discussion. Other key individuals will be included as authors or contributors as appropriate, at the discretion of the Senior Management Group (SMG). Any disputes relating to authorship will be resolved by the Steering Committee.

The Chair and Independent members of the Steering Committee will be acknowledged, but will not qualify for full authorship, in order to maintain their independence. Individual collaborators must not publish data concerning their participants' which are directly relevant to the questions posed in the study until the main results of the study have been published.

Conclusion

This research will deliver the first research-supported, best-for-patient, joint-specific, cost-effective recommendations for follow-up pathways, providing a gold standard for clinical excellence, and follow-up advice for patients, surgeons, purchasers and the NHS as a whole. Value is not limited to the UK, but has substantial global impact potential.

The impact of this work will be to reduce the burden on patients and the NHS in terms of outpatient visits and clinical tests that do not add benefit, while optimising detection of potential problems. From an NHS perspective, this work will provide managers with economic and clinical information on arthroplasty follow-up to inform service planning and delivery, and the role of arthroplasty practitioners in this service, with the potential to reduce geographical disparity through NHS trusts modelling their service provision on a national evidence-based guideline; provide orthopaedic surgeons with guidance on follow-up, including patient and economic considerations of factors involved; produce arthroplasty follow-up guidelines for adoption by the relevant specialist

For peer review only

societies and information for their members. From a patient perspective, this work will help to inform patients about follow-up practice, empower them to make choices about future healthcare relating to their joint arthroplasty and provide reassurance that their follow-up pathway is appropriate

The outputs of this project, in terms of evidence-based support for timing of follow-up and identification of the most cost-effective follow-up model, fit directly within the NHS framework for improving outcomes from elective procedures. Rationalising current diversity of follow-up practices should enable substantial savings for the NHS. We envisage outputs to be readily applicable to the wider NHS, not only hip and knee but also other joint replacements. With the committed support of key national and international organisations already in place, we anticipate that these guidelines will be positively received and that implementation will be widespread.

For peer review only

References

1. National Joint Registry for England, Wales, Northern Ireland and the Isle of Man: 15th Annual report 2018 http://www.njrreports.org.uk/; 2018.

2. Vanhegan IS, Malik AK, Jayakumar P, et al. A financial analysis of revision hip arthroplasty: the economic burden in relation to the national tariff. *J Bone Joint Surg Br* 2012;94(5):619-23.

3. Briggs T. Getting it right first time. Improving the quality of orthopaedic care within the National Health Service in England2012.

4. Singh JA. Epidemiology of Knee and Hip Arthroplasty: A Systematic Review. *The Open Orthopaedics Journal* 2011;5:80-5.

5. British Orthopaedic Association. Primary total hip replacement: a guide to good practice. 2012.

6. Smith LK. A Survey of the Current State of Hip Arthroplasty Surveillance in the United Kingdom. *Musculoskeletal Care* 2014;12(4):232-8.

7. Robert G, Harlock J, Williams I. Implementation Science. 2014;9:123.

8. Higgins JPT GSe. Cochrane Handbook for Systematic Reviews of Interventions Version 5.1.0 [updated March 2011]. The Cochrane Collaboration, 2011. Available from http://handbook.cochrane.org.

9. Liberati A, Altman DG, Tetzlaff J, et al. The PRISMA Statement for Reporting Systematic Reviews and Meta-Analyses of Studies That Evaluate Health Care Interventions: Explanation and Elaboration. *PLOS Medicine* 2009;6(7):e1000100.

10. Higgins JP, Altman DG, Sterne JA. Chapter 8: Assessing risk of bias in included studies in Cochrane handbook for systematic reviews of interventions Version 5.1.0: Available from http://handbook.cochrane.org.; 2011.

11. Wells G, Shea B, O'Connell D, et al. The Newcastle-Ottawa Scale (NOS) for assessing the quality of nonrandomised studies in meta-analyses

http://www.ohri.ca/programs/clinical_epidemiology/oxford.asp; 2014.

12. Dixon-Woods M, Cavers D, Agarwal S, et al. Conducting a critical interpretive synthesis of the literature on access to healthcare by vulnerable groups. *BMC Med Res Methodol* 2006;6(1):35.

13. Drummond MF, Schulpher MJ, Claxton K, et al. Methods for the Economic Evaluation of Health Care Programmes (4th edition). Oxford: Oxford University Press; 2015.

14. http://www.cprd.com

15. http://www.researchone.org

16. http://www.hscic.gov.uk/hes.

17. http://www.njrcentre.org.uk.

18. http://www.hscic.gov.uk/hesproms.

19. Pinedo-Villanueva R, Turner D, Raftery JP, et al. Outcomes after total hip replacement. *Osteoarthritis Cartilage* 2014;22:S214.

For peer review only

20. Pinedo-Villanueva R, Turner D, Raftery JP, et al. Primary care costs attributable to osteoarthritis before total hip replacement. *Osteoarthritis and Cartilage* 2014;22:S210-S1.

21. Jason PF, Gray RJ. A proportional hazards model for the subdistribution of a competing risk. *Journal of the American Statistical Association* 1999;94(446):496-509.

22. Prieto-Alhambra D, Pages-Castella A, Wallace G, et al. Predictors of fracture while on treatment with oral bisphosphonates: a population-based cohort study. *J Bone Miner Res* 2014;29(1):268-74.

23. Hirsch K, Wienke A. Software for semiparametric shared gamma and log-normal frailty models: An overview. *Comput Methods Programs Biomed* 2012;107(3):582-97.

24. Peduzzi P, Concato J, Kemper E, et al. A simulation study of the number of events per variable in logistic regression analysis. *J Clin Epidemiol* 1996;49(12):1373-9.

25. Wallace G, Judge A, Prieto-Alhambra D, et al. The effect of body mass index on the risk of post-operative complications during the 6 months following total hip replacement or total knee replacement surgery. *Osteoarthritis Cartilage* 2014;22(7):918-27.

26. Burn E, Liddle AD, Hamilton TW, et al. Cost-effectiveness of unicompartmental compared with total knee replacement: a population-based study using data from the National Joint Registry for England and Wales. *BMJ Open* 2018;8(4):e020977.

27. Fitzpatrick R, Shortall E, Sculpher M, et al. Primary total hip replacement surgery: a systematic review of outcomes and modelling of cost-effectiveness associated with different prostheses. *Health Technol Assess* 1998;2(20):1-64.

28. https://www.nice.org.uk/process/pmg9/chapter/foreword.

29. Basu A, Rathouz PJ. Estimating marginal and incremental effects on health outcomes using flexible link and variance function models. *Biostatistics* 2005;6(1):93-109.

30. Clarke P, Leal J, Kelman C, et al. Estimating the cost of complications of diabetes in Australia using administrative health-care data. *Value Health* 2008;11(2):199-206.

31. Mullahy J. Much ado about two: reconsidering retransformation and the two-part model in health econometrics. *J Health Econ* 1998;17(3):247-81.

32. https://www.nice.org.uk/about/what-we-do/our-programmes/nice-guidance/nice-technology-appraisal-guidance.

For peer review only

Authors' contributions

CCM - contributed to the planning and design of this study, drafted the article, revised the article and approved the final manuscript.

SRK - contributed to the planning and design of this study, drafted the article, revised the article and approved the final manuscript.

NA - contributed to the planning and design of this study, revised the article and approved the final manuscript

JH - contributed to the planning and design of this study, revised the article and approved the final manuscript

AJ - contributed to the planning and design of this study, revised the article and approved the final manuscript

JM - contributed to the planning and design of this study, revised the article and approved the final manuscript

JO - contributed to the planning and design of this study, revised the article and approved the final manuscript

RPV - contributed to the planning and design of this study, revised the article and approved the final manuscript

LKS - contributed to the planning and design of this study, revised the article and approved the final manuscript

CS - contributed to the planning and design of this study, revised the article and approved the final manuscript

CMT - contributed to the planning and design of this study, revised the article and approved the final manuscript

RW - contributed to the planning and design of this study, revised the article and approved the final manuscript

JMW - contributed to the planning and design of this study, revised the article and approved the final manuscript

PGC - contributed to the planning and design of this study, revised the article and approved the final manuscript

MS - contributed to the planning and design of this study, revised the article and approved the final manuscript

Funding statement

For peer review only

For peer review only - http://bmjopen.bmj.com/site/about/guidelines.xhtml

This article presents independent research funded by the National Institute for Health Research (NIHR) Health Services and Delivery Research Programme (14/70/146) and by the NIHR Leeds Biomedical Research Centre (BRC).

Disclaimer

The views expressed are those of the author(s) and not necessarily those of the NIHR or the Department of Health and Social Care.

Competing interests

None declared.

For peer review only

Appendix 1
Ovid MEDLINE(R) Epub Ahead of Print, In-Process & Other Non-Indexed Citations, Ovid MEDLINE(R) Daily and Ovid MEDLINE(R) 1946 to Present
Search Strategy:
----
1 Arthroplasty, Replacement, Knee/ (18061)
2 Knee Prosthesis/ (10316)
3 (TKA or TKR or UKR).tw. (9131)
4 Arthroplasty, Replacement, Hip/ (21968)
5 Hip Prosthesis/ (21263)
6 (THA or THR).tw. (30477)
7 or/1-6 (79707)
8 Hip/ (11074)
9 Osteoarthritis, Hip/ (7521)
10 Hip Joint/ (24959)
11 Hip?.tw. (121242)
12 Femur Head/ (8758)
13 Acetabulum/ (9886)
14 ("Femur head*" or "femoral head*" or acetabul*).tw. (27939)
15 "Total joint".tw. (4508)
16 Knee/ (12862)
17 Knee Joint/ (47967)
18 Osteoarthritis, Knee/ (15086)
19 Knee?.tw. (122420)
20 or/8-19 [Knee or Hip joints] (259908)
21 Joint Prosthesis/ (9772)
22 "Prostheses and Implants"/ (43103)
23 (Arthroplast* or replace* or implant* or prosthes* or unicompartment*).tw. (724712)
24 (Surf* or resurf*).tw. (990965)
25 or/21-24 [Arthroplasty] (1680546)
26 and/20,25 (82595)
27 7 or 26 [Hip or Knee Arthoplasty] (113853)
28 Longitudinal studies/ (109550)
29 Prospective studies/ (457925)
30 Time/ or time factors/ (1126356)
31 Follow-up studies/ (586823)
32 Epidemiological Monitoring/ (5660)
33 or/28-32 [Follow-up Studies MeSH] (2051292)
34 Retreatment/ (7424)
35 Reoperation/ (76777)
36 Treatment failure/ (31517)
37 exp Postoperative Complications/ (482686)
38 exp Prosthesis failure/ (25670)
39 or/34-38 [Complications MESH] (555690)
40 Risk factors/ (699996)
41 33 or 39 or 40 [Long term complications or risks MESH] (2963609)
42 *Postoperative Care/ (14831)
43 Postoperative care/mt (9905)
44 Postoperative Period/ (42528)
45 Aftercare/ (7484)
46 or/42-45 [Post Operative Care MeSH] (69319)
47 41 and 46 [Post op follow up MeSH] (29208)
48 Critical Pathways/ (5783)
49 47 or 48 [Post op follow up or pathways MeSH] (34925)

For peer review only

50 (("After care" or aftercare or "after surgery" or "after arthroplas*") adj3 follow-up).tw. (2471)
51 ((Postoperati* or post-operati* or "post surger*" or "post arthroplast*") adj3 follow-up).tw. (13670)
52 ((pathway* or care or treatment* or appointment* or consultation*) adj3 follow-up).tw. (29535)
53 ((Postoperati* or post-operati* or "post surger*" or "post arthroplast*") adj3 (surveillance* or monitor*)).tw. (4101)
54 or/50-53 [Follow-up studies Textword] (49052)
55 (("After care" or aftercare or "after surgery" or "after arthroplas*") adj3 (failur* or reoperat* or re-operat* or readmission or readmit* or revision or revisions)).tw. (691)
56 ((Postoperati* or post-operati* or "post surger*" or "post arthroplast*") adj3 (failure* or reoperat* or re-operat* or readmission or readmit* or revision or revisions)).tw. (5650)
57 ((pathway* or care or treatment* or appointment* or consultation* or follow-up or time or risk*) adj8 (revis* adj2 surgery)).tw. (1505)
58 or/55-57 [Post op complications Textword] (7776)
59 (("After care" or aftercare or "after surgery" or "after arthroplas*") adj3 risk*).tw. (637)
60 ((Postoperati* or post-operati* or "post surger*" or "post arthroplast*") adj3 risk).tw. (12221)
61 or/59-60 [Post op risks Textword] (12801)
62 (("After care" or aftercare or "after surgery" or "after arthroplas*") adj3 pathway*).tw. (93)
63 ((Postoperati* or post-operati* or "post surger*" or "post arthroplast*") adj3 pathway*).tw. (181)
64 (Care pathway* or clinical pathway* or critical pathway*).tw. (6772)
65 or/62-64 [Post op pathways Textword] (6956)
66 54 or 58 or 61 or 65 [Post op follow up Textword] (75424)
67 49 or 66 [Post Op Follow Up] (106039)
68 27 and 67 [TJA Post op follow up] (3634)

For peer review only
