# Supplementary material for: Towards UK poSt Arthroplasty Follow-up rEcommendations (UK SAFE): protocol for an evaluation of the requirements for arthroplasty follow-up, and the production of consensus-based recommendations
Source: BMJ Open. 2019 Jun 25;9(6):e031351. doi: 10.1136/bmjopen-2019-031351 (PMC6597629; doi:10.1136/bmjopen-2019-031351)
Supplement: Reviewer comments [file bmjopen-2019-031351.reviewer_comments.pdf]

PEER REVIEW HISTORY

BMJ Open publishes all reviews undertaken for accepted manuscripts. Reviewers are asked to complete a checklist review form (**http://bmjopen.bmj.com/site/about/resources/checklist.pdf**) and are provided with free text boxes to elaborate on their assessment. These free text comments are reproduced below.

ARTICLE DETAILS

**TITLE (PROVISIONAL)**	Towards UK poSt Arthroplasty Follow-up rEcommendations (UK SAFE): protocol for an evaluation of the requirements for arthroplasty follow-up, and the production of consensus-based recommendations
**AUTHORS**	Czoski Murray, Carolyn; Kingsbury, Sarah; Arden, Nigel; Hewison, Jenny; Judge, Andrew; Matu, Jamie; O'Shea, Jamie; Pinedo-Villanueva, Rafael; Smith, Lindsay; Smith, Chris; Thomas, Christine; West, Robert; Wright, Judy; Conaghan, Philip; Stone, Martin

This article was not externally reviewed at BMJ Open. Protocols that have been independently assessed prior to submission to BMJ Open are usually fast-tracked to publication on the grounds that further substantial changes will not be possible. This independent assessment will usually be external, independent review for both a substantial grant award from a non-commercial or government funder and ethics approval.
